# Ticks, Fleas, and Harboured Pathogens from Dogs and Cats in Cyprus

**DOI:** 10.3390/pathogens11121403

**Published:** 2022-11-23

**Authors:** Anastasia Diakou, Dimitra Sofroniou, Barbara Paoletti, Androniki Tamvakis, Stanislav Kolencik, Dimitris Dimzas, Simone Morelli, Marika Grillini, Donato Traversa

**Affiliations:** 1 Laboratory of Parasitology and Parasitic Diseases, School of Veterinary Medicine, Faculty of Health Sciences, Aristotle University of Thessaloniki, 54124 Thessaloniki, Greece; 2 Independent Researcher, 25088 Lemesos, Cyprus; 3 Faculty of Veterinary Medicine, Teaching Veterinary Hospital, University of Teramo, 64100 Teramo, Italy; 4 Laboratory of Ecology and System Dynamics, Department of Marine Sciences, University of the Aegean, 81100 Mytilene, Greece; 5 Department of Biology, University of Nevada Reno, Reno, NV 89557, USA; 6 Department of Animal Medicine, Production and Health, University of Padua, 35020 Legnaro, Italy

**Keywords:** ectoparasites, epidemiology, pet animals, vector-borne pathogens

## Abstract

Ticks and fleas are blood-sucking ectoparasites that cause irritation and anaemia to their hosts and act as vectors of pathogens (vector-borne pathogens, VBPs) of relevance for animal and human health. In the present study, tick and flea species in dogs and cats from Cyprus were recorded and VBPs were detected in the collected specimens. Ectoparasites were collected from 220 animals (161 dogs and 59 cats), and a questionnaire including demographic, clinical, and other information was filled out for each animal. The ectoparasites were morphologically identified and the detection of VBPs was performed by PCR-coupled sequencing. *Rhipicephalus sanguineus* sensu lato was found on 108 dogs and 13 cats, and *Ixodes gibbosus* on 2 dogs. *Ctenocephalides felis* was the predominant flea species (on 62 dogs and 45 cats), while one dog and one cat were infested by *Ctenocephalides canis* and *Echidnophaga gallinacea*, respectively. The VBPs in ticks were *Anaplasma platys*, *Rickettsia massiliae*, *Rickettsia conorii*, *Rickettsia felis*, *Hepatozoon felis* and *Hepatozoon canis*, while *Rickettsia felis*, *Rickettsia* sp., *Bartonella koehlerae*, *Bartonella clarridgeiae*, and *Bartonella henselae* were recorded in fleas. Statistical analysis (chi-square test and multiple univariate generalized linear model) showed that animals up to 6 months of age were less likely to be infested with ticks than older animals, but more likely to be infested with fleas. Ticks were more prevalent in sheltered than in owned animals, while the odds ratio of flea presence was higher in owned animals than those living in shelters. The present study is the first investigation on the occurrence of ticks and fleas in dogs and cats from Cyprus, showing the presence of different VBPs in these important ectoparasites. The results point out the importance of systematic ectoparasite control in dogs and cats.

## 1. Introduction

Ticks and fleas are blood-sucking arthropods, infesting several vertebrates, among them dogs and cats. They have been extensively studied because of their direct clinical impact on animals, the pathogens they transmit, and their relevance in human health [1,2]. These ectoparasites can cause discomfort and may severely impact the health and well-being of dogs and cats. Ticks cause nuisance, anaemia, irritation, cutaneous lesions with inflammation and eosinophilic aggregation, secondary infections occasionally leading to abscesses or even pyaemia, and toxicosis (tick paralysis). Fleas cause severe irritation, pruritus and self-wound formation, blood loss and anaemia, and flea-associated allergic dermatitis [3,4,5]. Ticks and fleas may also transmit various vector-borne pathogens (VBPs) to their hosts, many of which are zoonotic. Pathogens transmitted by ticks to dogs and cats include mostly protozoa (e.g., *Babesia* spp., *Hepatozoon* spp., *Cytauxzoon* spp.) and bacteria (*Rickettsia* spp., *Ehrlichia* spp., *Anaplasma* spp., *Coxiella* spp., *Borrelia* spp.). Fleas are vectors of *Bartonella* spp., *Rickettsia felis*, and *Yersinia pestis*, and are also an intermediate host of the cestodes *Dipylidium caninum* and *Hymenolepis diminuta*, and the nematode *Acanthocheilonema reconditum* [3,6,7,8]. 

Specific drivers, e.g., climate change and global warming, destruction of wild habitats for agriculture intensification, landscape modification, poor ecosystem protection, and increase in pet travel have a significant impact on the epidemiology and the increasing occurrence of ectoparasites [6]. Consequently, the affiliated VBPs and associated diseases are expected to expand, emerge, or re-emerge in many areas [9]. Knowledge of the current epidemiology of ticks, fleas, and transmitted pathogens is still scant in many areas of Europe and their distribution and occurrence are constantly changing over time [10]. 

In Cyprus, some investigations on ticks and tick-borne pathogens have been conducted in the past [11,12,13,14,15,16], while data on fleas and flea-borne pathogens are limited to only rats, foxes, and hares [14,17,18]. Therefore, the aim of the present study was (i) to investigate the infestation by ticks and fleas in dogs and cats from Cyprus; (ii) to detect the presence of VBPs in these ectoparasites; and (iii) to associate findings with different possible risk factors, in order to update and enrich knowledge about the epidemiology of these important ectoparasites.

## 2. Materials and Methods

### 2.1. Animals and Ectoparasite Collection

**Figure 1:**
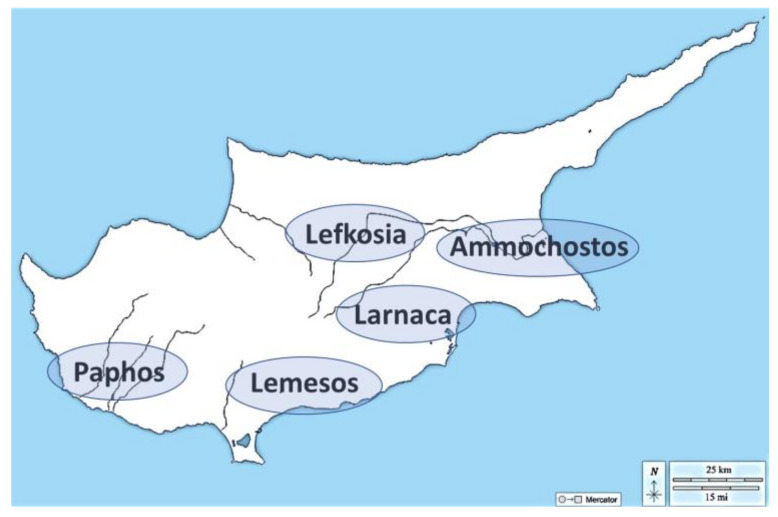
The map of Cyprus and the districts from which the sampled animals originated.

The survey was conducted on 220 animals (161 dogs and 59 cats), living in five districts of Cyprus, i.e., Ammochostos, Larnaca, Lemesos, Lefkosia, and Paphos (Figure 1), and presented to a private veterinary clinic in Limassol for routine clinical examinations (e.g., vaccination, castration, investigation of clinical condition, injury). Ectoparasites were detected by fur and skin inspection and by combing with a stainless-steel flea comb. The ectoparasites were collected by entomological forceps, stored in Eppendorf tubes containing 70° ethanol, and tagged with an individual code. For each animal included in the survey, a questionnaire was filled out, with information about age, sex, country district, lifestyle, last ectoparasiticide administration, the reason for the visit, and clinical and laboratory findings.

### 2.2. Identification of Ectoparasites

The collected ectoparasites were transferred to the Laboratory of Parasitology and Parasitic Diseases, School of Veterinary Medicine of the Aristotle University of Thessaloniki. The ectoparasites were examined under a stereomicroscope (8×–64×) and a light microscope (100×, 400×) for identification based on their morphological characteristics [19,20,21].

### 2.3. Detection of VBPs

After identification, ectoparasite specimens were transferred to the Laboratory of Parasitology of the Faculty of Veterinary Medicine, University of Teramo, for the detection of VPBs by molecular methods.

Ticks and fleas were examined in pooled samples per animal, into groups of one to five individuals. Overall, 122 pooled tick samples and 111 pooled flea samples were examined, excluding highly engorged tick specimens to avoid excess nucleic acids of vertebrate host origin. The ectoparasite pools were homogenized before DNA extraction. Briefly, the specimens were taken from the 70° ethanol solution, air-dried, and mechanically crushed in a 1.5 mL safe-lock tube with sterile pestles. The homogenates were incubated with proteinase K solution overnight at 56 °C and total nucleic acids were extracted from these homogenates in accordance with the manufacturer’s instructions (Exgene Tissue SV, Gene All, South Korea). In ticks, *Anaplasma* spp./*Ehrlichia* spp., *Babesia* spp., *Bartonella* spp., *Rickettsia* spp., and *Hepatozoon* spp., and in fleas, *Bartonella* spp. and *Rickettsia* spp., were detected by polymerase chain reaction (PCR). A fragment of the 18S rRNA gene of *Anaplasma*/*Hepatozoon* spp. and *Babesia* spp., a partial sequence of the 16S–23S rRNA intergenic species region (ITS) of *Bartonella* spp., and a fragment of the rickettsial outer membrane protein A (ompA) gene were amplified using primers and protocols described previously [22,23,24,25]. The primers used for the amplification of the targeted DNA are shown in Table 1. All amplifications included a positive control containing genomic target DNA and a negative control without DNA. PCR products were visualized under UV illumination after electrophoresis migration on a 1.8% agarose gel. PCR products were sequenced in one direction, using the same primers as those used for DNA amplification. Sequences were compared for similarity to sequences in GenBank, using the BLAST program hosted by NCBI, National Institutes of Health, USA (http://www.ncbi.nlm.nih.gov, accessed on 1 August 2022).

### 2.4. Statistical Analysis

The occurrence of fleas and ticks on dogs and cats was evaluated in relation to factors expressing demographic details (gender, age), status (owned or sheltered), and previous treatments (time passed since the last dosing). Moreover, the existence of VBPs in the ectoparasites was associated with additional factors: geographic region, the status of the animal (owned or sheltered), and clinical examination or laboratory findings associated with disease (e.g., anorexia, weight loss, eye lesions, neurological signs, positive in-clinic diagnostic test for infectious diseases). The chi-square test of independence was used to assess the effect of the above factors on the occurrence of ectoparasites and the existence of VBPs, respectively. The significant factors defined by the chi-square test were then entered into a multiple univariate generalized linear model (GLM) for determining their combined effect on the occurrence of ectoparasites [26]. The odds ratios with their corresponding confidence intervals (C.I.) were used to compare the proportion of the occurrence of each ectoparasite among the factor groups. The information collected through the questionnaires, about the veterinary product used on some of the animals, was not included in the statistical analysis owing to missing or unreliable data. The statistical analysis was implemented using the R package version [27] and the Rcmdr package [28].

## 3. Results

### 3.1. Study Animals 

The demographics and other details of the examined animals are shown in Table 2.

### 3.2. Ectoparasites

From a total of 161 dogs with ectoparasites, 98 and 51 had ticks or fleas only, respectively, while 12 had mixed tick and flea infestation. Accordingly, in total, 110 (68.3%) dogs were infested with ticks and 63 (39.1%) had fleas, including both single and mixed infections. From a total of the 59 cats infested with ectoparasites, 9 had only ticks; 45 had only fleas; 3 had ticks and fleas; 1 had fleas and lice; and 1 had a mixed infestation with ticks, fleas, and lice. In total, 13 (22%) cats were infested with ticks, 50 (84.7%) with fleas, and 2 (3.4%) with lice, including both single and mixed infections (Table 3 and Table 4).

Two different tick species were identified: *Rhipicephalus sanguineus* sensu lato (s.l.) on 108 dogs and 13 cats, and *Ixodes gibbosus* on 2 dogs. The most abundant flea species was *Ctenocephalides felis*, found on 62 dogs and 45 cats, while *Ctenocephalides canis* and *Echidnophaga gallinacea* were found on one dog and one cat, respectively. The mixed infestations included 10 dogs and 4 cats with *R. sanguineus* s.l. and *C. felis*; two dogs with *I. gibbosus* and *C. felis*; one cat with *R. sanguineus* s.l., *C. felis*, and the louse *Felicola subrostratus*; and one cat infested with *E. gallinacea* and *F. subrostratus* (Table 4).

### 3.3. Detection of VBPs

In total, 233 ectoparasite samples (122 tick and 111 flea samples) were examined for the detection of VBPs. In the case of multiple ticks or flea specimens per animal, a pooled sample (per ectoparasite type and per animal) was prepared. VBPs’ detection by PCR was not possible for one tick and two flea samples owing to an insufficient or not suitable DNA sample. Overall, 32 (14.5%) animals were infested with ectoparasites that harboured one or more VBPs, whereas 35 (15%) ectoparasite pool samples were positive for VBPs, because, in three cases (two dogs and one cat) with a mixed infestation by *R. sanguineus* s.l. and *C. felis*, VBPs were found in both ticks and fleas.

The DNA of six different pathogens was detected in ticks, i.e., *Anaplasma platys*, *Rickettsia massiliae*, *Rickettsia conorii*, *Rickettsia felis*, *Hepatozoon felis*, and *Hepatozoon canis*, while no *Babesia* spp. was found in the examined specimens. The DNA of five different VBPs was detected in fleas, i.e., *Rickettsia felis*, *Rickettsia* sp., *Bartonella koehlerae*, *Bartonella clarridgeiae,* and *Bartonella henselae*. Details about the species and number of animals in the ectoparasites of which these VBPs were detected are shown in Table 5. 

Sequencing of PCR products and BLAST analysis revealed similarities of the herein detected VBPs with DNA sequences published in GenBank, as shown in Table 6.

### 3.4. Statistical Analysis

Chi-square test of independence showed that neither tick nor flea presence was related to the time passed since the last ectoparasitic treatment (*χ^2^* = 3.68, df = 4, *p* > 0.05 for ticks and *χ^2^* = 3.54, df = 4, *p* > 0.05 for fleas) or the animal’s sex (*χ^2^* = 0.60, df = 1, *p* > 0.05 for ticks and *χ^2^* = 0.02, df = 1, *p* > 0.05 for fleas). On the other hand, the occurrence of ectoparasites was associated with the age of the host (*χ ^2^* = 27.19, df = 3, *p* < 0.001 for ticks and *χ^2^* = 20.90, df = 3, *p* < 0.001 for fleas) and their “owned or sheltered” status (*χ^2^* = 14.99, df = 1, *p* < 0.001 for ticks and *χ^2^* = 16.34, df = 1, *p* < 0.001 for fleas) (Table 7).

The investigation of the association between VBPs’ occurrence and various factors showed that VBPs’ detection was not associated with clinical signs or findings of disease (*χ^2^* = 2.42, df = 1, *p* > 0.05), the animals’ “owned or sheltered” status (*χ^2^* = 1.06, df = 1, *p* > 0.05), or the region of living (*χ^2^* = 3.62, df = 4, *p* > 0.05) (Table 8).

The age category and the “owned or sheltered” status were further analysed for their combined effect on the occurrence of ticks or fleas using multiple GLM (Table 9). The analysis showed that animals up to 6 months and those between 6 and 12 months had the same likelihood to be infested by ticks or fleas (multiple GLM *p*-values > 0.05). However, young animals had a higher likelihood of being infested with fleas, whereas older animals had a higher likelihood of being infested with ticks. Indeed, animals up to 6 months were 0.26 and 0.27 times less likely to be infested with ticks than animals 1 to 7 years or older, respectively. Animals up to 6 months were 3.59 and 4.88 times more likely to be infested with fleas than animals from 1 to 7 years and those older than 7 years, respectively (multiple GLM *p*-value < 0.01). The status (owned or sheltered) of the animal was also found to be related to the presence of ectoparasites (multiple GLM *p*-value < 0.01). Ticks were five times (i.e., the inverse of 0.2 odds ratio shown in Table 9) more likely to be found on sheltered animals than owned animals, while the odds ratio of flea presence was 4.84 times higher in owned animals than in those living in shelters. 

## 4. Discussion

Cyprus, an island country in the Eastern Mediterranean Sea, is a cosmopolitan hub and a centre of tourism, market, education, and other activities, which receives a great number of visitors throughout the year. On the other hand, Cyprus has a large number of dogs and cats, living as owned pets, free-roaming, or strays. A significant number of animal shelters in the country are actively facilitating adoption of stray animals, which, in many cases, travel abroad, to their new home, in different areas of the world. In this context, investigating and monitoring pathogens that may be transmitted locally or in remote countries via ticks and fleas is of great epidemiological importance.

The subtropical–Mediterranean climate of Cyprus with mild winters and warm to hot summers is favourable to ticks and fleas, because their development, especially the rate of transition from one development stage to the next, which in most cases takes place in the environment, is temperature-dependent [29,30]. The present results are in line with the fact that tick parasitism is more common in dogs than in cats, while the opposite is true for flea infestations, probably because of the different behaviour of dogs and cats and the different biology of these ectoparasites [1,31].

The ectoparasite species identified herein have a worldwide distribution and are prevalent in Southern Europe [32]. The predominant tick species, *R. sanguineus* s.l. [33], also made up the majority (89–92%) of the ticks collected from dogs in earlier surveys in Cyprus, showing limited affiliation to other host species (mouflons, foxes, hares, goats, sheep, and bovines) [13,15]. It is a three-host tick, a fact that facilitates the transmission of VBPs from animal to animal and is the vector of many VBPs [4,34]. Accordingly, 6 different VBPs were detected in 22 out of 120 *R. sanguineus* s.l. samples examined in the present study. 

The prevalence of *A. platys*, the agent of canine cyclic thrombocytopenia (CCT), varies between 0.4% and 87.5% in different areas of the world [35]. In Cyprus, this bacterium has been detected only once in a dog [16]. *Anaplasma platys* is a recognized zoonotic agent [35], and enriching information on its occurrence in areas where data are lacking is important. The present results confirm that this VBP is circulating among ticks and dogs in Cyprus.

Even though seropositive dogs to *R. conorii* are highly prevalent in southern Europe [9,36,37,38,39], usually, only a small number of the examined ticks score positively in PCR [40,41,42], which is consistent with the present results. The infection in dogs is usually subclinical, but in humans, *R. conorii* is the agent of Mediterranean spotted fever [43], thus creation of epidemiological information is essential. Interestingly, *R. massiliae* was the most prevalent VBP in the present study. It is considered an emerging pathogen in Africa, Europe, and the USA, incriminated for several human cases with clinical signs similar to Mediterranean spotted fever [44]. On the basis of the present findings, *R. massiliae* is a possible emerging public health threat in Cyprus and the awareness towards this bacterium should be increased.

Both *H. canis* and *H. felis* were found in ticks, albeit at a low prevalence. In Cyprus, *H. canis* has been previously reported in dogs [45], while *H. felis* has been detected with a high prevalence (37.9%) in cats [46]. Similarly, *H. felis* occurs with a high prevalence in cats in other European enzootic areas, reaching 25.5% in Greece [47]. *Hepatozoon* species circulating in Europe, i.e., *H. canis* in dogs and *H. canis*, *H. felis*, and *Hepatozoon silvestris* in cats, have diverse pathogenic potentials. Although infections are often subclinical, animals may develop severe disease depending on the species or haplotype involved [47,48,49,50]. 

To the best of the authors’ knowledge, this is the first record of *I. gibbosus* on dogs in Cyprus. It is one of the most common *Ixodes* species on the island [15,31] and it was previously reported on mouflons [51]. *Ixodes gibbosus* is adapted to warm and dry climates, replacing *Ixodes ricinus* in the eastern Mediterranean, which is less resistant to such conditions [31]. Further investigations into the prevalence and vectorial capacity of *I. gibbosus*, as the dominant *Ixodes* species in the area, would be of merit.

*Ctenocephalides felis* is the vector of important pathogens, including *B. henselae* detected herein and previously reported in rats and cats of Cyprus [6,46,52]. On the other hand, to the best of the authors’ knowledge, the present report of *B. koehlerae* and *B. clarridgeiae* is the first in the country. *Bartonella* spp. are agents of disease in both animals and humans; for example, *B. henselae* is the agent of cat-scratch disease [53], thus constant surveillance of the presence of these VBPs in dogs, cats, and ectoparasites is pivotal.

*Rickettsia felis* is the agent of human flea-borne spotted fever and an emerging VBP [6]. In Cyprus, it has been detected previously in *C. felis* from rats [17]. The cat flea is the primary vector of *R. felis*, but it is probably also transmitted by other flea species, ticks, and other blood-sucking arthropods [6,54] and it was also detected in *R. sanguineus s.l.* in the present study.

*Ctenocephalides canis*, the dog flea, is less common in dogs than *C. felis* [6]. Accordingly, this flea species was found only on one dog in the present study. Nevertheless, in some areas, *C. canis* is reported to be more prevalent than *C. felis* [55]. The dog flea may also transmit pathogens including *R. felis* and *B. henselae*; however, because of its limited abundance compared with the cat flea, its vectorial role is considered inferior [56]. 

The flea *E. gallinacea*, also known as the “sticktight flea”, was found on one cat. This species is common on fowl, but it also infests mammals, most frequently cats, probably owing to bird hunting [57]. It is a flea species of both veterinary and medical importance, transmitting fowl viruses, *Y. pestis*, *R. typhi*, and *D. caninum* [58], which renders it an important target for study and control, despite its low frequency.

An incidental finding in the examination for ticks and fleas was the cat louse *F. subrostratus* on two cats. Cat louse has a worldwide distribution and infestation is often an indication of a poor general health condition and lack of care [59]. Even if out of the scope of the present article, this finding is important as the cat louse has been identified as a potential intermediate host of a *Dipylidium* species, genetically distant from *D. caninum*, infecting hyenas, dogs, and cats [60]. 

The use of ompA gene appears to be specific and discriminating for the spotted fever group *Rickettsiae*, but some authors recommend that multiple gene targets should be used to gain an accurate identification [61]. This could be the reason that, for a few isolates, identification only to the genus level was feasible (Table 5). The remainder of VBPs identified in the present study showed a varying level of similarity with GeneBank deposits, isolated from different hosts and areas of the world (Table 6). It is worth noting that the detection of *Rickettsia* spp. DNA, mainly in *R. sanguineus*, provides evidence that this tick may be among the main vectors of *Rickettsia* spp. in Cyprus, according to previous studies [40,62]. 

The finding that young animals (<6 months) were significantly less likely to be infested with ticks, but more likely to be infested with fleas, may be attributed to the fact that young animals will spend most of their time in a restricted environment near their home, in close proximity to their mother and siblings, a condition that favours host-to-host flea transmission [1]. This contrasts with older animals that spend more time roaming a wider area outdoors. As such, older animals are more likely to come into contact with ticks, explaining the finding that older animals were significantly more likely to harbour ticks than young animals.

The activity within a wider or restricted environment may also be the reason ticks were more prevalent on sheltered animals, especially considering that some of them were introduced recently and were previously roaming in a wider area of their region. Accordingly, the occurrence of fleas was more frequent in owned animals, living in a confined/restricted environment (indoors for most cats, indoors or/and in the garden for dogs), which can often maintain flea infestation, compared with those living in shelters. 

Interestingly, the infestation was not statistically associated with the time that had passed since the last ectoparasitic application. Thus, animals with a recent ectoparasite treatment were at the same risk of infestation as the rest of the animals. Although drug resistance development in ectoparasites is a known problem [29,63], the lack of specific investigation into the products used and the application practices does not allow further evaluation of this finding.

## 5. Conclusions

Ticks and fleas are a major concern for pet owners, veterinarians, and medical doctors because of their clinical impact on dogs and cats and the VBPs they transmit. The results of the present study provide new knowledge about the occurrence of ticks and fleas in dogs and cats from Cyprus, and the pathogens that these ectoparasites may transmit, covering a relevant gap in knowledge. Companion animals travelling for adoption (commonly sheltered animals) or with their owners for vacations may facilitate the spreading of VPBs [45]. This risk is lurking, particularly in animal movements from and to Mediterranean areas, including Cyprus, as this part of Europe is considered a major epidemiological hub for VBPs [47]. Systematic ectoparasite control is pivotal and a plethora of veterinary products are available for this purpose. Furthermore, the research into new animal and environment-friendly tools for control is ongoing, and effective biological or botanical-based compounds and vaccines may also be available in the future [64,65].

## Figures and Tables

**Table 1:** Primers used for the detection of VBPs in ectoparasites of dogs and cats from Cyprus and corresponding references (Ref).

Primer	Pathogen	Target Gene	Nucleotide Sequences (5′-3′)	Product Size (bp)	Ref.
Rrl9O.70p	*Rickettsia*	190 kDa antigen	ATGGCGAATATTTCTCCAAAA	~532	[22]
Rrl9O.602n			AGTGCAGCATTCGCTCCCCCT		
325s	*Bartonella*	16S-23S rRNA ITS	CTTCAGATGATGATCCCAAGCCTTYTGGCG	~600	[23]
1100as			GAACCGACGACCCCCTGCTTGCAAAGCA		
Piro A	*Babesia*	18S rRNA	AATACCCAATCCTGACACAGGG	400	[24]
Piro B			TTAAATACGAATGCCCCCAAC		
EHR16SD	*Anaplasma/Ehrlichia*	18S rRNA	GGTACCYACAGAAGAAGTCC	345	[24]
EHR16SR			TAGCACTCATCGTTTACAGC		
Tabar F	*Hepatozoon*	18S rRNA	CCAGCAGCCGCGGTAATTC	373	[25]
Tabar R			CTTTCGCAGTAGTTYGTCTTTAACAAATCT		

**Table 2:** Recorded data for the dogs and cats (n = 220) with ectoparasites examined in Cyprus.

	Factor	Dogs (n = 161)	Cats (n = 59)
Status	Owner/Shelter	134/27	51/8
Region	Lefkosia	32	3
	Lemesos	91	42
	Larnaca	28	6
	Paphos	8	4
	Amochostos	2	4
Sex	Male/Female	81/80	23/36
Age	<6 months	23	17
	6–≤12 months	13	14
	>1–≤7 years	93	25
	>7 years	32	3
Last treatment for ectoparasites	≤1 month	17	2
	1–≤3 months	17	6
	>3–≤6 months	11	3
	>6–≤12 months	22	0
	>12 months	94	48
Reason for visit or findings	Disease/Other	88/73	34/25

**Table 3:** Number (n) of animals (dogs or cats) in Cyprus, infested with different types of ectoparasites, with the corresponding confidence interval (C.I.) of the occurrence percentage.

Animal Species (Sample Size)	Ticks n (%C.I.)	Fleas n (%C.I.)	Ticks and Fleas n (%C.I.)	Fleas and Lice n (%C.I.)	Ticks, Fleas, and Lice n (%C.I.)
Dogs (n = 161)	98 (60.8 ± 7.7)	51 (31.7 ± 6.7)	12 (7.5 ± 3.1)	0	0
Cats (n = 59)	9 (15.3 ± 7.0)	45 (76.3 ± 12.2)	3 (5.1 ± 3.3)	1 (1.7 ± 1.4)	1 (1.7 ± 1.4)

**Table 4:** Species identification of ticks and fleas and mixed infections in dogs and cats from Cyprus.

Animal Species (Sample Size)	*Rhipicephalus sanguineus* s.l.	*Ixodes gibbosus*	*Ctenocephalides felis*	*Ctenocephalides canis*	*Echidnophaga gallinacea*
Dogs (n = 161)	108 ^1^	2 ^2^	62 ^1,2^	1	0
Cats (n = 59)	13 ^3,4^	0	45 ^3,4^	0	1 ^5^

^1^ Ten dogs with mixed infestation by *R. sanguineus* and *C. felis*; ^2^ 2 dogs with mixed infestation by *I. gibbosus* and *C. felis*; ^3^ 4 cats with mixed infestation by *R. sanguineus* and *C. felis*; ^4^ a cat with a mixed infestation by *R. sanguineus*, *C. felis*, and *Felicola subrostratus*; ^5^ a cat with a mixed infestation by *E. gallinacea* and the louse *F. subrostratus*.

**Table 5:** Vector-borne pathogens (VBPs) detected in 122 tick and 111 flea pooled samples (per ectoparasite type and per animal) collected from dogs and cats in Cyprus.

Animal Species	VBPs in Ticks						VBPs in Fleas				
	*A. p*	*R. m*	*R. c*	*R. f*	*H. c*	*H. f*	*R. f*	*R.* sp.	*B. k*	*B. c*	*B. h*
Dogs (n)	3	10	1	-	3	1	3	4	1	2	-
Cats (n)		2	-	1	-	1	5	-	-		1
Total	3	12	1	1	3	2	8	4	1	2	1

n = number of animals in the ectoparasites of which the pathogen was found, *A. p.* = *Anaplasma platys*; *R. m* = *Rickettsia massiliae*; *R. c* = *Rickettsia conorii*; *R. f* = *Rickettsia felis*; *H. f* = *Hepatozoon felis*; *H. c* = *Hepatozoon canis*; *R*. sp. = *Rickettsia* sp.; *B. k* = *Bartonella koehlerae*; *B. c* = *Bartonella clarridgeiae*; *B. h* = *Bartonella henselae*.

**Table 6:** Vector-borne pathogens (VBPs) detected in ticks and fleas from dogs and cats in Cyprus, and their similarity with GenBank entries.

VBP (n of Sequences Analyzed)	GenBank Accession Number	Similarity
*Anaplasma platys* (n = 3)	JX392984.1	99%
*Rickettsia massiliae* (n = 12)	MW026209.1	97–99%
*Rickettsia felis* (n = 9)	KP318094.1	96–99%
*Hepatozoon felis* (n = 2)	KY649442.1	100%
*Hepatozoon canis* (n = 3)	MK645969.1	97–100%
*Rickettsia conorii* (n = 1)	AE006914.1	97%
*Rickettsia* sp. (n = 4)	MF134884.1	96–99%
*Bartonella koehlerae* (n = 1)	MT095046.1	98%
*Bartonella clarridgeiae* (n = 2)	EU589237.1	96%
*Bartonella henselae* (n = 1)	KT314216.1	100%

**Table 7:** Chi-square test of independence showing associations between the occurrence of ectoparasites and various factors recorded for each animal.

	Ticks			Fleas		
Variable	Positive	Negative	*p*-Value	Positive	Negative	*p*-Value
**Last treatment**			0.451			0.471
≤1 month	14 (73.7%)	5 (26.3%)		6 (31.6%)	13 (68.4%)	
>1–3 months	12 (52.2%)	11 (47.8%)		13 (56.5%)	10 (43.5%)	
>3–6 months	6 (42.9%)	8 (57.1%)		8 (57.1%)	6 (42.9%)	
>6–12 months	13 (59.1%)	9 (40.9%)		11 (50.0%)	11 (50.0%)	
>12 months	78 (54.9%)	64 (45.1%)		75 (52.8%)	67 (47.2%)	
**Sex**			0.438			0.875
Male	61 (58.7%)	43 (41.3%)		54 (51.9%)	50 (48.1%)	
Female	62 (53.4%)	54 (46.6%)		59 (50.9%)	57 (49.1%)	
**Age category**			0.000 *			0.000 *
<6 months	13 (32.5%)	27 (67.5%)		30 (75.0%)	10 (25.0%)	
6–12 months	7 (25.9%)	20 (74.1%)		20 (74.1%)	7 (25.9%)	
>1–7 years	81 (68.6%)	37 (31.4%)		49 (41.5%)	69 (58.5%)	
>7 years	22 (62.9%)	13 (37.1%)		14 (40.0%)	21 (60.0%)	
**Status**			0.000 *			0.000 *
Owned	93 (50.3%)	92 (49.7%)		106 (57.3%)	79 (42.7%)	
Sheltered	30 (85.7%)	5 (14.3%)		7 (20.0%)	28 (80.0%)	

* Statistically significant factor, *p* < 0.001.

**Table 8:** Contingency tables with chi-square test results between VBPs’ existence and other factors.

	VBPs		*p*-Value
Variable	Positive	Negative	
**Signs/findings**			0.120
Disease	21 (17.8%)	97 (82.2%)	
Other	10 (10.3%)	87 (89.7%)	
**Status**			0.304
Owned	24 (13.3%)	156 (86.7%)	
Sheltered	7 (20.0%)	28 (80.0%)	
**Region**			0.460
Ammochostos	2 (40.0%)	3 (60.0%)	
Larnaca	3 (9.4%)	29 (90.6%)	
Lemesos	18 (13.7%)	113 (86.3%)	
Lefkosia	6 (17.1%)	29 (82.9%)	
Paphos	2 (16.7%)	10 (83.3%)	

**Table 9:** Assessment of risk factors of ectoparasites’ occurrence including the results of the multiple generalized linear model (GLM).

	Ticks			Fleas		
Variable	Odds Ratio	95% CI	GLM *p*-Value	Odds Ratio	95% CI	GLM *p*-Value
**Age category**						
<6 m vs. 6–12 m	1.40	(0.47, 4.43)	0.557	1.05	(0.32, 3.30)	0.928
vs. 1–7 years	0.26	(0.11, 0.55)	0.001 *	3.59	(1.61, 8.54)	0.002 *
vs. >7 years	0.27	(0.10, 0.67)	0.007 *	4.88	(1.83, 13.82)	0.001 *
**Status**						
Owned vs. Sheltered	0.20	(0.06, 0.52)	0.002 *	4.84	(2.04, 12.91)	0.001 *

* Multiple GLM *p*-value < 0.01, identifying a risk factor.

## Data Availability

Not applicable.
